# Invasive and anticoagulant treatment for coronary ectasia: a single operator’s experience in a tertiary hospital in South Africa

**Published:** 2009-08

**Authors:** V Grigorov

**Affiliations:** ARW YP Medical Centre and Glynwood Hospital, Johannesburg, South Africa

## Abstract

**Introduction:**

Coronary artery ectasia (CAE) is a rare but well-recognised condition involving dilatation of a coronary artery to more than 1.5 times the diameter of the adjacent portion of the artery. As far as we are aware, the disease has not been described in any local literature and no other research has been conducted in Africa. We carried out this research in order to establish the incidence of the condition in South Africa, as well as the possible preferred method of treatment.

**Methods:**

Cases were identified from the database of the practice. The study involved only patients who were classified to have Markis type I, II and III disease. From a total of approximately 2 000 angiographies performed during the study period, CAE types I, II and III were diagnosed in 20 patients. Patients with type IV CAE were excluded from the group. Nineteen patients were male and were in their fourth or fifth decade of life, and one was female. Three of the patients were Indian, one was black, and the rest were white.

**Results:**

Four patients in the group had diabetes, 13 were smokers and eight had hypertension. Dyslipidaemia was observed in seven patients. The most commonly affected area of the coronary artery was the RCA-19 (isolated, or in combination). Patients were treated mainly with anticoagulation and, when necessary, with angioplasty and stent implantation. Morbidity was seen in 5% of the patients and no mortality was observed.

**Conclusion:**

Most of the patients were male, and the occlusion involved mostly the right coronary artery. The cause of occlusion is still unknown, but it is thought to be due to slow blood flow, damaged endothelium, or a combination of the two. The best therapeutic approach is not known as yet.

## Summary

Coronary artery ectasia (CAE) is a dilatation of a segment of a coronary artery to at least 1.5 times the diameter of the adjacent segment.^1^ The finding of coronary ectasia is uncommon but has been described in the literature. The cause of the disease remains obscure, but atherosclerosis is thought to be the major culprit. The best therapeutic approach is not known as yet.

Bourgon originally described the condition in 1812,^2^ but Bjork gave it the term ectasia.^3^ Nowadays ‘coronary ectasia’ and ‘coronary aneurysm’ are used interchangeably for the condition. The first classification was that of Markis in 1976,^4^ based on vessel involvement: type I – diffuse ectasia of two or more major vessels, type II – diffuse ectasia in one vessel and localised disease in another, type III – diffuse ectasia in one vessel only, and type IV – localised involvement only.

There are few reviews and articles published elsewhere on the condition, and to our knowledge only one report from South Africa.^5^ The largest body of research describing the condition is the CASS registry,^6^ showing a 4.9% incidence from the 20 000 angiograms they reviewed.

Cases of coronary ectasia are generally related to atherosclerosis, although they are sometimes linked to conditions such as scleroderma, Ehler-Danlos, polyarteritis nodosa and Kawasaki disease.^7^,^8^ Occasionally, large ulcerated coronary plaques can be misinterpreted on angiogram and though to be aneurysms. Usually their true appearance is seen via intravascular ultrasound (IVUS).^9^ CAE is seen more frequently in patients with abdominal and ascending aorta aneurysms.^10^

Although the cause of CAE formation is unknown, the histopathological characteristics of the disease are similar to the changes observed in coronary atherosclerosis, and there are theories that they may be linked.^11^ In the pathological process of formation of coronary ectasia, extensive media degeneration and hyalinisation is suspected, possibly as a result of chronic vascular inflammation.^12^ Over-stimulation with nitric oxide is another hypothesis proposed by Sorrell.^13^

It is important to note the distinct differences between poststenotic dilatation of an artery and coronary ectasia, as they have different mechanisms of formation.

## Methods

The cases of coronary ectasia were identified via the database. We included patients with Markis type I, II and III disease, as diagnosed from their angiographic recordings. CAE was defined as a dilatation of the coronary artery diameter to at least 1.5 times that of the normal adjacent segment. Coronary occlusion was defined as a no-flow region behind the affected segment and coronary stenosis as a narrowing of more than 50% of the coronary lumen. Hypertension was defined as values above 140/90 mmHg, diabetes as fasting blood glucose values above 8 mmol/l, and hypercholesterolaemia as serum total cholesterol above 5.2 mmol/l and low-density lipoprotein cholesterol (LDL-C) above 2.4 mmol/l.

## Results

**Fig. 1.**
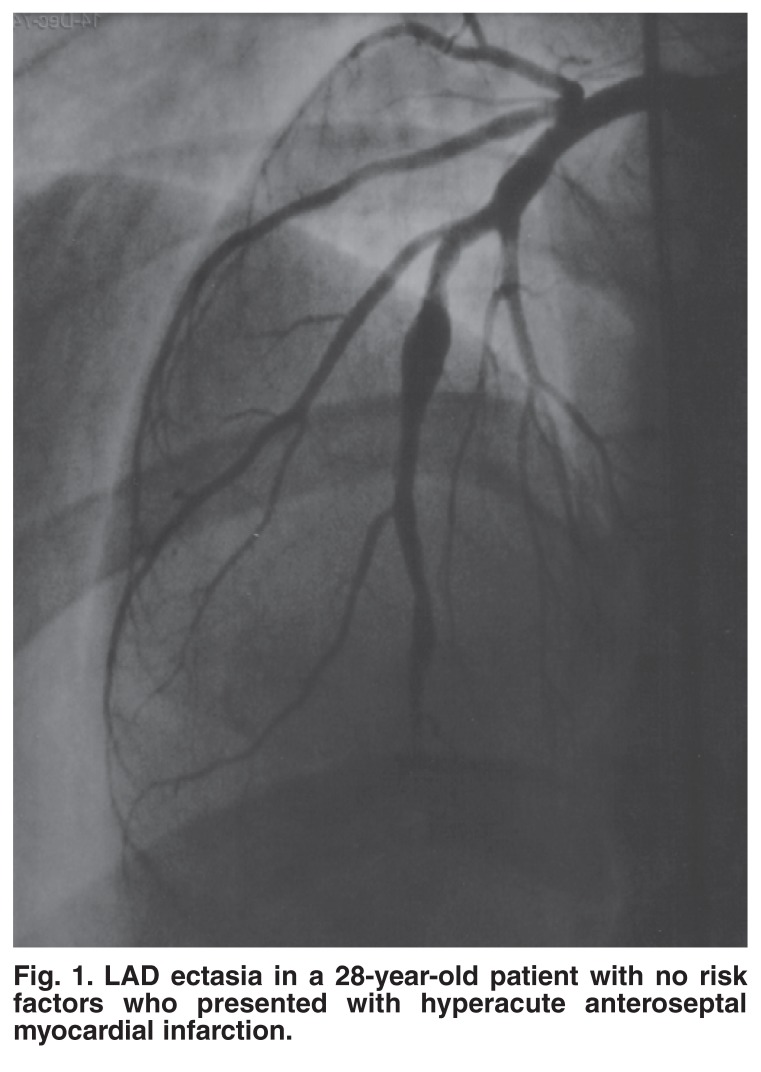
LAD ectasia in a 28-year-old patient with no risk factors who presented with hyperacute anteroseptal myocardial infarction.

**Fig. 2.**
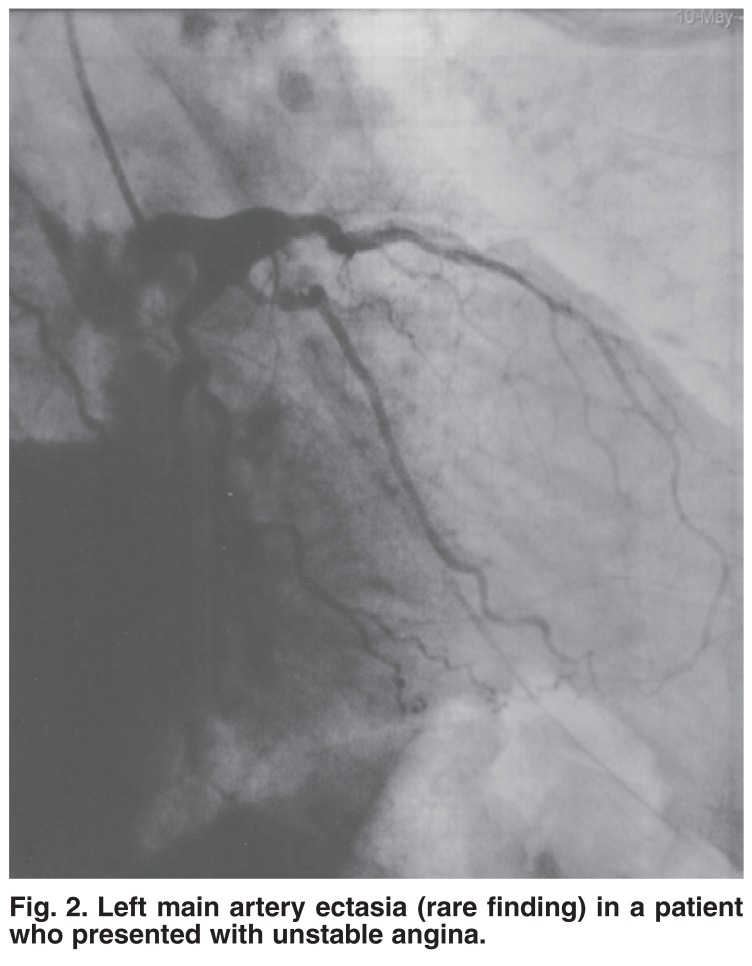
Left main artery ectasia (rare finding) in a patient who presented with unstable angina.

From approximately 2 000 angiographies performed in the selected study period, we found 20 patients with coronary ectasia Markis type I, II and III (an incidence of 1%). (Markis type IV was not included in the study, as we did not think it warranted aggressive anticoagulant management.) Figs 1 and 2 give examples of coronary ectasia.

Of the 20 patients, 19 were males and one was female. They presented as follows: stable angina in nine (45%) patients, unstable angina or non-STEMI in five (25%), and STEMI in six (30%). The median age of the patients with CAE was 50 years and the mean was 51 years (range 28–65). Almost all were males (95%). Of the population, 60% were white, 35% were Indian/coloured, and 5% were black (Table 1).

**Table 1:** Characteristics Of The Group

	*n*	*%*
Males	19	95
Female	1	5
Stable angina	9	45
Non-STEMI or unstable angina	5	25
STEMI	6	30
Median age	50	
Mean age	51	
White	12	60
Indian/coloured	7	35
Black	1	5
Family history of CAD	7	35
Diabetes mellitus	4	20
Hypertension	8	40
Dyslipidiamia	7	35
Co-existent obstructive CAD	11	55

Approximately 2 000 coronary angiograms – 20 patients in study

There was a family history was of CAD in seven (35%) white patients. Diabetes was present in 20%, hypertension in 40%, and dyslipidaemia in 35% of the patients. Co-existent obstructive coronary artery disease was present in 11 of the cases (55%) (Table 1). The frequency of arterial involvement was as follows: right coronary artery (RCA) in 95% of the patients, left anterior descending artery (LAD) in 65%, circumflex artery (Cx) in 50%, Markis type I in nine (45%), Markis type II in six (30%) and Markis type III in five (25%) patients (Tables 2, 3). Based on the angiographic finding, none of the patients were referred for surgery, 10 (50%) had angioplasty with or without a stent, and the rest had medical therapy only (Table 4).

**Table 2:** Arterial Involvement

*Artery*	*%*
RCA	95
LAD	65
Circumflex	50

**Table 3:** Breakdown Of Patients According To Markis Type

*Markis class*	*n*	*%*
Type I	9	45
Type II	6	30
Type III	5	25

**Table 4:** Patient Management

*Management*	*n*	*%*
Surgery		0
Angioplasty and stent	10	50
Medical treatment	10	50
Clexane only	7	35
Clexane and Methylase	2	10
IIb IIIa inhibitor	9	45

**Fig. 3.**
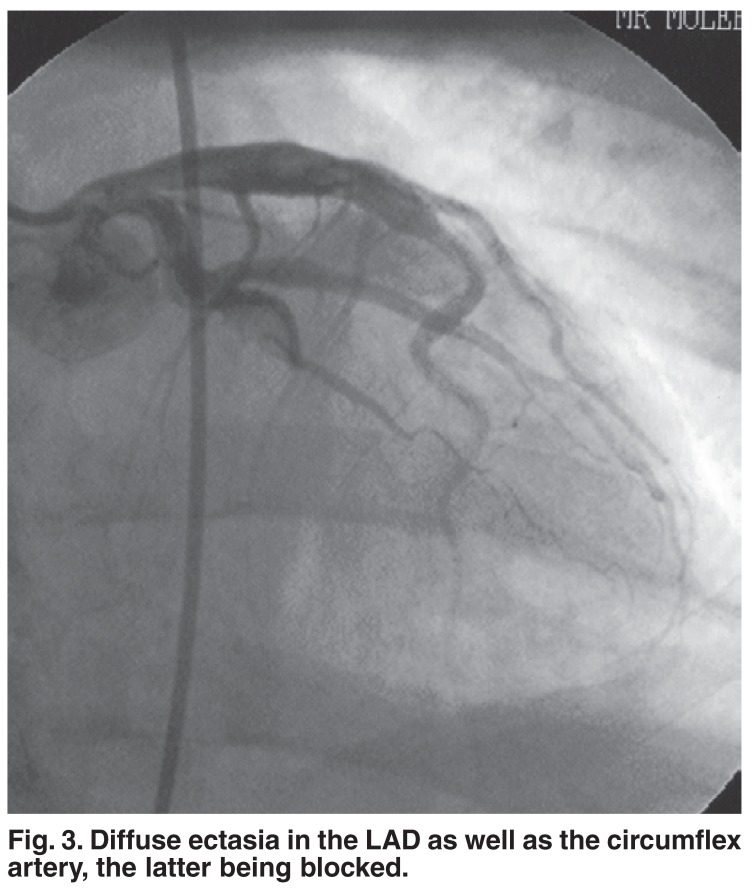
Diffuse ectasia in the LAD as well as the circumflex artery, the latter being blocked.

**Fig. 4.**
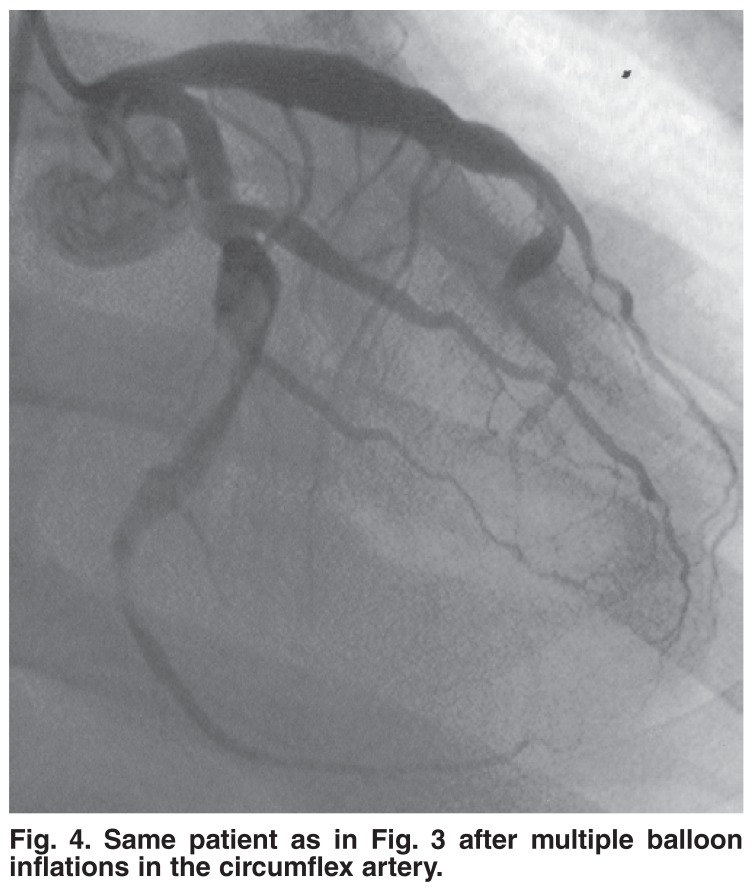
Same patient as in Fig. 3 after multiple balloon inflations in the circumflex artery.

Seven (35%) patients were treated with Clexane alone, and two (10%) had Clexane in combination with Methylase. A IIb/IIIa antagonist was used in nine (45%) of the cases. Figs 3 and 4 show a patient who was treated with Intergrilin and subsequently had angioplasty.

There were no recorded deaths in the study period. The mean follow-up period was 4.2 years. Most of the patients (15; 75%), irrespective of the approach, were started on warfarin. Two were still considering their options (patient indecisiveness: 10%). The patients in the group who received a stent (non-DES) were also on Plavix for three months. All patients on warfarin had a controlled prothrombin index (PI) between 30 and 50% (Table 5).

**Table 5:** Outcome Of Follow-Up Period

*Outcome*	*n*	*%*
Mean follow up: 4.2 years		
Patients on warfarin	15	75
Nitrate	3	15
Beta-blocker	15	75
Calcium channel blocker	1	5
Aspirin	20	100
ACE inhibitor	7	35
Mortality	0	0
Morbidity	1	5

Nitrate was given in three patients (15%), β-blockers in 15 (75%), calcium channel blockers in one (5%), aspirin in all (100%), and ACE inhibitors in seven (35%). The mortality for the period of follow up was zero (0%) and the morbidity was one (5%) (Table 5).

## Discussion

To our knowledge, this is the first reported series of coronary ectasia in South Africa. Furthermore, these are the first reported results of the management of coronary artery ectasia Markis types I, II and III treated predominantly with warfarin.^13^,^14^ The incidence of coronary ectasia was similar to that seen in other studies.^10^,^14^,^15^ Our slightly smaller number compared with other studies was probably due to the size of our sample, as well as the fact that patients with Markis type IV were not included.

A significant gender difference was observed with coronary ectasia, probably because the presence of coronary artery disease is much less frequent in females than in males. The most commonly involved artery in CAE is the RCA, although for unknown reasons.^10^,^14^ The least affected artery appears to be the left main artery (LMA), possibly because in coronary artery disease, the LMA is usually least affected.

The prognosis for CAE is also not clear, as the published literature does not provide specific data. Regarding management and therapy, there are several opinions. Some studies, which are based on significant flow disturbances within the ectatic segments, suggest anticoagulation should be the main therapy.^13^,^14^ IVUS management, with or without bare or covered stent positioning, has been recommended with caution, as the effects still have to be proven.^16^ In our group of patients there were no deaths. The patients were reviewed on a six-monthly basis, and had their PI monitored fortnightly.

A limitation of this research was that there was no control group. In this study no patients underwent bypass surgery. This could possibly be considered if there is a large thrombus or aneurysm. 17 Only 50% of our cases had percutaneous transluminal coronary angioplasty (PTCA) alone, and/or had a stent. The rest were managed with warfarin as well as statins (if the cholesterol level was above 5.2 mmol/l), diabetic and antihypertensive medication, and some received Plavix. The whole group received additional aspirin therapy of 160 mg/day.

Judging by the literature, CAE does not appear to be a benign disease. It has been observed that a narrowing can occur adjacent to an area of ectasia.^18^ Coronary ectasia is prone to spasm.^19^ Dissections and thromboses are well reported. During angiography we observed that almost all patients had reduced TIMI frame count and other authors have reported similar results.^20^ With long-term follow up, it has been seen that patients with coronary ectasia do not fare any better or worse than patients with coronary obstructive disease.^21^,^22^

Stenosis is not the only way in which coronary atherosclerosis presents. Ectasia is sometimes observed in isolation or in combination with stenosis. Probably the word ‘remodeling’ should be used rather than ‘stenosis’ when one speaks of coronary atherosclerosis.^23^

This brings one to the question of what medication patients should use. In this study we tried not to use nitrates due to the perception that they can further enlarge the already dilated artery and lead to more ischaemia. We managed all the risk factors and employed standard use of warfarin for life. We are not sure if the combination of treatment or the warfarin itself contributed to the low percentage of adverse events on the patients’ follow-ups.

The reason we decided to enroll only patients with Markis type I, II and III disease in the study was that we deemed it logical in these patients to use long-term warfarin therapy. The results of the patients on warfarin were not compared with the rest where no warfarin was used (i.e. Markis type IV), as we felt the groups were incomparable in terms of the severity of the disease.

## Conclusion

The observed incidence of CAE in this study coincided with international findings. Most of the patients were male, and most had involvement of the right coronary artery. The incidence in males was much higher compared with females. CAE does not appear to be a benign disease. It is still unknown what causes the occlusion of the ectatic vessel – slow blood flow, damaged endothelium, or a combination of the two.

Strict PI control in this group led to zero percent complications over the follow-up period. Nevertheless the period was too short and the group too small to suggest a therapeutic approach.
